# Drying kinetic of industrial cassava flour: Experimental data in view

**DOI:** 10.1016/j.dib.2017.10.008

**Published:** 2017-10-07

**Authors:** Oluwole A. Odetunmibi, Oluyemisi A. Adejumo, Pelumi E. Oguntunde, Hilary I. Okagbue, Adebowale O. Adejumo, Esivue A. Suleiman

**Affiliations:** a Department of Mathematics, Covenant University, Ota, Nigeria; b AIDE Department, National Centre for Agricultural Mechanization, Ilorin, Nigeria; c Department of Statistics, University of Ilorin, Ilorin, Nigeria

**Keywords:** Drying rate, Drying time, Drying kinetic, Industrial cassava flour, Temperature, Velocity, Dewatering, Moisture content, Grated unfermented cassava mash

## Abstract

In this data article, laboratory experimental investigation results on drying kinetic properties: the drying temperature (*T*), drying air velocity (*V*) and dewatering time (Te), each of the factors has five levels, and the experiment was replicated three times and the output: drying rate and drying time obtained, were observed. The experiment was conducted at National Centre for Agricultural Mechanization (NCAM) for a period of eight months, in 2014. Analysis of variance was carried out using randomized complete block design with factorial experiment on each of the outputs: drying rate and drying times of the industrial cassava flour. A clear picture on each of these outputs was provided separately using tables and figures.

It was observed that all the main factors as well as two and three ways interactions are significant at 5% level for both drying time and rate. This also implies that the rate of drying grated unfermented cassava mash, to produce industrial cassava flour, depend on the dewatering time (the initial moisture content), temperature of drying, velocity of drying air as well as the combinations of these factors altogether. It was also discovered that all the levels of each of these factors are significantly difference from one another. In summary, the time of drying is a function of the dewatering time which was responsible for the initial moisture content. The higher the initial moisture content the longer the time of drying, and the lower the initial moisture content, the lower the time of drying. Also, the higher the temperature of drying the shorter the time of drying and vice versa. Also, the air velocity effect on the drying process was significant. As velocity increases, rate of drying also increases and vice versa. Finally, it can be deduced that the drying kinetics are influenced by these processing factors.

**Specification Table**

**Table:** 

Subject area	Engineering and Bio-system
More specific subject area	Post harvest, Food process
Type of data	Tables and figures
How data was acquired	Unprocessed secondary data
Data format	Laboratory experimental investigation results on temperature, drying air velocity, dewatering (initial moisture content), and the outputs (drying rate and drying time of industrial cassava flour)
Experimental factors	Temperature, drying air velocity, dewatering (initial moisture content)
Experimental features	Computational analysis: Analysis of variance (ANOVA), Randomized complete block design with factorial experiment (5^3^), Histogram
Data source location	National Centre for Agricultural Mechanization (NCAM), Idofian, Ilorin, Nigeria.
Data accessibility	All the data are in this data article as a Supplementary data file
Software	SPSS Statistical program and Microsoft Excel

**Value of the data**

● The data on drying rate of industrial cassava flour will be useful for the industries that are planning to embark on large production of cassava flour.
● The data on drying time of industrial cassava flour will be useful also for the industries to know the time needed for the cassava flour to dry at different levels of temperature and velocity.
● The data can be useful for the quality assurance of any cassava flour processing company.
● The data will be useful in food processing, post harvest and bio-system engineering studies.
● The data can be useful for educational purposes and nutrition assessment studies.
● The data is useful in the study of drying kinetic of food processing into powdered particle.
● Several known statistics, for example, root mean squared error (RMSE), linear regression, and complete randomized design (CRD) can be applied which provides alternatives to analysis with randomized complete block design (RCBD) with factorial experiment used in this paper.

## 1 Data

The data describes the kinetic properties of industrial cassava flour. The raw data which consist of 375 rows, with each row having five columns: dewatering, temperature, velocity, and two outputs (drying time and drying rate) can be assessed as Supplementary data.

Statistical summary of the drying time and drying rate distributions of industrial cassava flour are presented in Table 1. It was observed that the average drying time of industrial cassava flour is 6088 s equivalent to 2 h: 9 min. The least and the highest drying time so far recorded in the experiment are 870 and 33,300 s respectively.

**Table 1:** Summary statistics of the drying time and drying rate distributions of the industrial cassava flour.

**Statistics**			
		Drying time	Drying rate
N	Valid	375	375
	Missing	0	0
Mean		6088.38	0.03667
Median		4800.00	0.01405
Mode		1980^a^	0.00923
Std. deviation		5553.273	0.05143
Variance		30,838,840.151	0.003
Skewness		2.330	1.656
Std. error of skewness		0.126	0.126
Kurtosis		7.681	1.308
Std. error of kurtosis		0.251	0.251
Minimum		870	0.00172
Maximum		33,300	0.18209
Percentiles	25	2160.00	0.00455
	50	4800.00	0.01405
	75	7830.00	0.03195

a Multiple modes exist. The smallest value is shown.

**Fig. 1:**
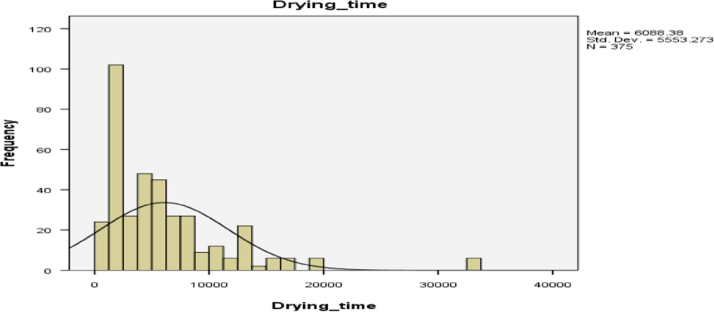
Drying time distribution of industrial cassava flour.

**Fig. 2:**
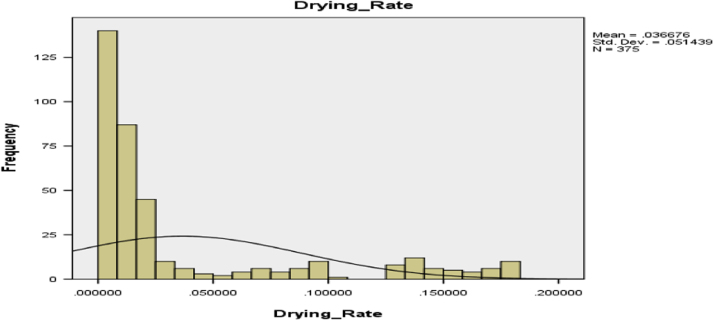
Drying rate distribution of industrial cassava flour.

Histogram for the drying time distribution is presented in Fig. 1. It can be observed from Fig. 1 that the Drying time distribution is not normally distributed, it skewed to right. The histogram for the Drying rate of industrial Cassava Flour is presented in Fig. 2, which also skewed to right.

## 2 Methods and materials

Randomized complete block design with factorial experiment (5^3^) was used for this analysis. The 5^3^ factorial experiment means three (3) factors (dewatering, temperature, and velocity) each at five (5) levels. The levels for dewatering are: (60 mins, 90 mins, 120 mins, 150 mins, 180 mins); temperature are: (40 °C, 55 °C, 70 °C, 85 °C, 100 °C) and air velocity are: (1.0 m/s, 1.5 m/s, 2.0 m/s, 2.5 m/s, 3.0 m/s). Each of these experiments was replicated three times. The total units of experiment were 5 × 5 × 5 × 3 which is 375 altogether. Analysis of variance (ANOVA) table was derived on each output.

Several studies have been conducted on the cassava [1], [2], [3], [4], [5], [6], [7], [8], [9], [10], [11], [12], [13], [14], [15], [16], [17], [18], [19], [20], [21]. Similar data articles on cassava that applied statistical tools can be helpful, readers are refer to [22], [23], [24].

Table 2 presents the analysis of variance results for drying time; it was observed that all the main factors as well as two and three ways interactions are significant at 5% level. This also implies that the time for grated unfermented cassava mash to get dry depends on the dewatering (the initial moisture content) of the grated unfermented cassava mash, temperature of the dryer, the air velocity of the machine as well as the combinations of these factors altogether.

**Table 2:** Analysis of variance for drying time.

Source	Type III sum of squares	DF	Mean square	*F*	Sig.
Corrected model	11,533,920,843.091^a^	124	93,015,490.670	31,344.591	0.000
Intercept	13,881,352,745.395	1	13,881,352,745.395	4,677,772.662	0.000
Dewatering	8,561,751,074.970	4	2,140,437,768.743	721,290.026	0.000
Temperature	760,368,437.328	4	190,092,109.332	64,057.710	0.000
Velocity	84,553,288.708	4	21,138,322.177	7123.244	0.000
Dewatering * Temperature	1,342,065,338.778	16	83,879,083.674	28,265.782	0.000
Dewatering * Velocity	195,116,311.236	16	12,194,769.452	4109.424	0.000
Temperature * Velocity	104,662,325.630	16	6,541,395.352	2204.336	0.000
Dewatering * Temperature * Velocity	435,720,716.436	64	6,808,136.194	2294.223	0.000
Error	741,878.333	250	2967.513		
Total	25,434,735,642.000	375			
Corrected total	11,534,662,721.424	374			

a R Squared = 1.000 (Adjusted R Squared = 1.000), Dependent Variable: drying time.

Table 3, Table 4, Table 5 present further tests, refer to as post hoc tests, to determine if there are differences in the mean results of levels of dewatering, temperature, and air velocity respectively. It was observed that all the levels of each of these factors are significantly different from one another. It was observed that the time of drying reduces with increase in the temperature levels. At the lowest temperature level 40 °C, the highest average time of drying was observed to be 2 hs: 57 min. This was followed by temperature level 55 °C with average drying time of 2 h: 22 min observed, then by 70 °C, and the average time of drying was 1 h: 47 min. At the next temperature level which is 85 °C, the time of drying was 1 h:30 min. The highest temperature level, which was 100 °C, the average time of drying was 1 h: 25 min.

**Table 3:** Post hoc test for significant differences in dewatering under drying time in seconds.

Dewatering	N	Subset				
		1	2	3	4	5
60 min	75					14,853.8800
90 min	75				6963.6000	
120 min	75			4816.2000		
150 min	75		2407.2000			
180 min	75	1400.4000

**Table 4:** Post hoc test for significant differences in temperature under drying time in seconds.

Temperature	N	Subset				
		1	2	3	4	5
40°	76					8632.0000
55°	74				6442.1467	
70°	75			5728.1333		
85°	75		5078.3108			
100°	75	4567.5000

**Table 5:** Post hoc test for significant differences in air velocity under drying time in seconds.

Velocity	N	Subset				
		1	2	3	4	5
1.0 m/s	75					6715.3333
1.5 m/s	75				6579.6000	
2.0 m/s	75			5938.2400		
2.5 m/s	75		5692.8133			
3.0 m/s	75	5515.2933				

**Fig. 3:**
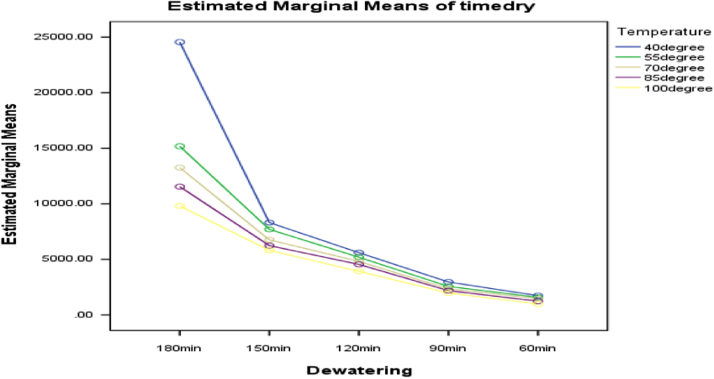
Graph of interactions between dewatering and temperature for drying time.

**Fig. 4:**
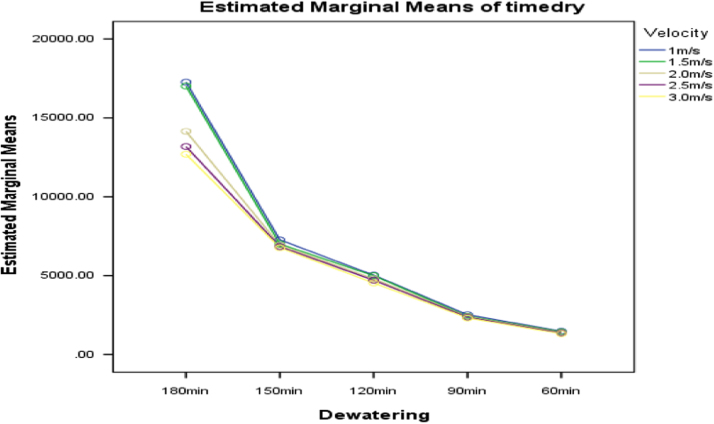
Graph of interactions between dewatering and air velocity for drying time.

**Fig. 5:**
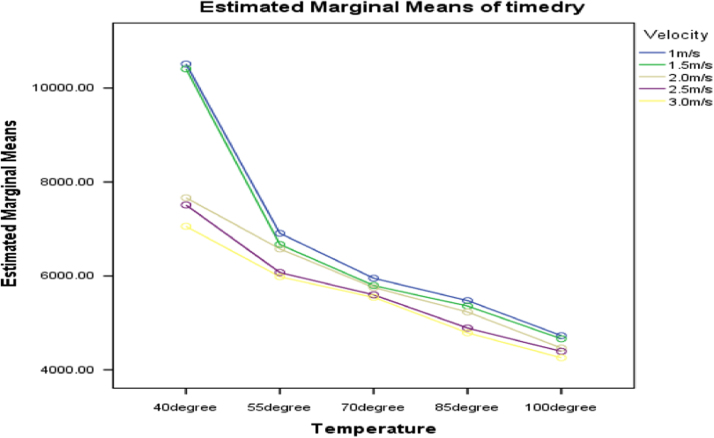
Graph of interactions between temperature and air velocity for drying time.

Fig. 3, Fig. 4, Fig. 5 present the graphs indicating the magnitude of interactions between each pair of these factors, that is, dewatering and temperature; dewatering and velocity; temperature and velocity respectively.

Likewise, Table 6 presents the analysis of variance results for drying rate. It was also observed that all the main factors as well as two and three ways interactions are significant at 5% level. This also implies that the drying rate of cassava flour depends on the dewatering (the initial moisture content) of the grated unfermented cassava mash, temperature of the dryer, the air velocity of the machine as well as the combinations of these factors combined.

**Table 6:** Analysis of variance for drying rate of industrial cassava flour.

Source	Type III sum of squares	Df	Mean square	F	Sig.
Corrected model	0.990^a^	124	0.008	8638.111	0.000
Intercept	0.503	1	0.503	544,968.220	0.000
Dewatering	0.886	4	0.221	239,727.010	0.000
Temperature	0.037	4	0.009	9915.007	0.000
Velocity	0.002	4	0.000	513.202	0.000
Dewatering * Temperature	0.059	16	0.004	4006.150	0.000
Dewatering * Velocity	0.003	16	0.000	197.766	0.000
Temperature * Velocity	0.000	16	2.850E-005	30.846	0.000
Dewatering * Temperature * Velocity	0.002	64	3.114E-005	33.707	0.000
Error	0.000	250	9.238E-007		
Total	1.494	375			
Corrected total	0.990	374			

a R Squared = 1.000 (Adjusted R Squared = 1.000), Dependent Variable: drying rate.

Table 7, Table 8, Table 9 present post hoc tests for dewatering, temperature, and air velocity respectively. Again it was observed that all the levels of each of these factors are significantly difference from one another. It was observed that drying rate of the grated unfermented cassava mash increases as the dewatering time increases at different air velocity levels of the drying process. In addition, the temperature increases with the drying rate of grated unfermented cassava mash.

**Table 7:** Post hoc test for significant differences in dewatering under drying rate.

Dewatering	N	Subset				
		1	2	3	4	5
60 min	75	0.0034711				
90 min	75		0.0057808			
120 min	75			0.0132115		
150 min	75				0.0285983	
180 min	75					0.1323260

**Table 8:** Post hoc test for significant differences in temperature under drying rate.

Temperature	N	Subset				
		1	2	3	4	5
40°	75	0.0231224				
55°	75		0.0299855			
70°	75			0.0366016		
85°	74				0.0422645	
100°	76					0.0512933

**Table 9:** Post hoc test for significant differences in air velocity under drying rate.

Velocity	N	Subset				
		1	2	3	4	5
1 m/s	75	0.0336577				
1.5 m/s	75		0.0350140			
2.0 m/s	75			0.0363696		
2.5 m/s	75				0.0384764	
3.0 m/s	75					0.0398699

**Fig. 6:**
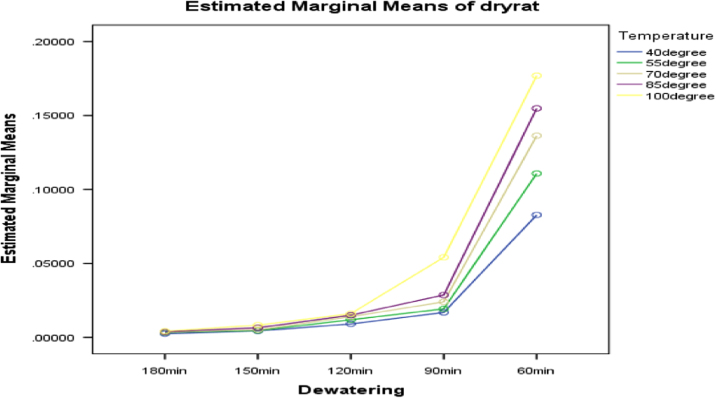
Graph of interactions between dewatering and temperature for drying rate.

**Fig. 7:**
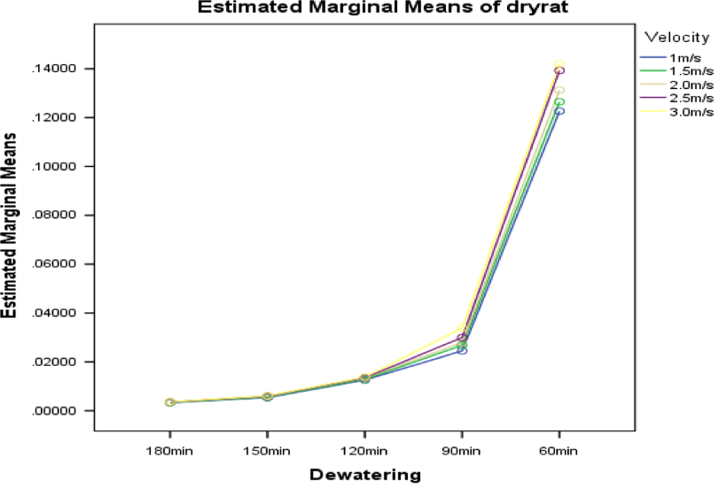
Graph of interactions between dewatering and air velocity for drying rate.

**Fig. 8:**
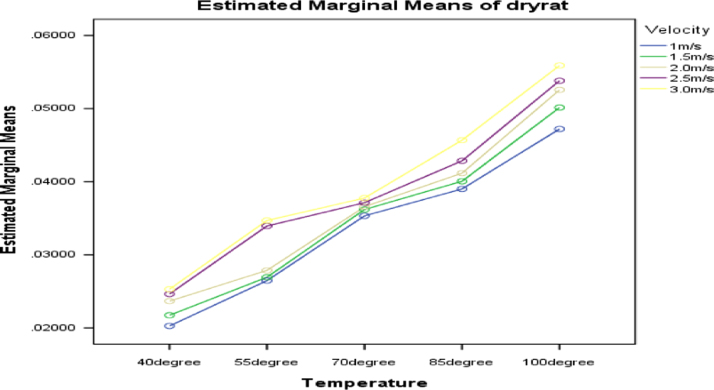
Graph of interactions between temperature and air velocity for drying rate.

Fig. 6, Fig. 7, Fig. 8 present the graphs indicating the level of interactions between each pair of these factors, that is, dewatering and temperature; dewatering and velocity; temperature and velocity respectively.

In summary, both the drying time and drying rate of any quantity of grated unfermented cassava mash depend on the initial moisture content (dewatering) of the mash, the temperature of the dryer, the air velocity of the machine and the effects of their interactions in pairs and the three factors altogether.
